# Supplementary material for: Salt-Mediated Organic Solvent Precipitation for Enhanced Recovery of Peptides Generated by Pepsin Digestion
Source: Proteomes. 2021 Nov 3;9(4):44. doi: 10.3390/proteomes9040044 (PMC8628918; doi:10.3390/proteomes9040044)
Supplement: Supplementary file 1 [file proteomes-09-00044-s001.zip › supplemental.pdf]

proteomes

(SUPPLEMENTAL MATERIAL)

Salt-mediated organic solvent precipitation for enhanced recovery of peptides generated by pepsin digestion

Venus Baghalabadi [1,2], Habib Razmi [1] and Alan Doucette [2,*]

[1] Department of Chemistry, Azarbaijan Shahid Madani University, Tabriz, Iran; venus.b@dal.ca (V.B); h.razmi@azaruniv.ac.ir (H.R.)
[2] Department of Chemistry, Dalhousie University, Halifax, Nova Scotia, Canada;
* Correspondence: alan.doucette@dal.ca; Tel.: 1-902-494-3414

Table of Contents

Page #

- Figure S1: Venn diagram of precipitated proteins – pellet *vs* supernatant……. 3
- Figure S2: BSA pepsin digests – effect of enzyme ratios………………………… 4
- Figure S3: BSA pepsin digests – effect of digestion time………………………… 5
- Figure S4: Comparison of peptide MW distribution in different digestion conditions ……………………………………………………………………………… 6
- Figure S5: Aggregated BSA sequence coverage………………………………….. 7

(additional files)

**** tables are presented as individual worksheets within the excel files***

- Table S1_S2.xls: List of peptides and proteins identified in the pellet (S1) and supernatant (S2) following salt-mediated solvent precipitation of pepsin-digested protein mixture
- Table S3_S4.xls: List of peptides and proteins identified in the pellet (S3) and supernatant (S4) following salt-mediated solvent precipitation of pepsin-digested yeast sample
- Table S5_S6.xls: List of peptides and proteins identified in the pellet (S5) and supernatant (S6) following salt-mediated solvent precipitation of pepsin-digested plasma sample
- Table S7_S8.xls: List of peptides and proteins identified in the pellet (S7) and supernatant (S8) following salt-mediated solvent precipitation of pepsin-digested alpha-casein sample
- Table S9_S10_S11.xls: List of BSA peptides identified in precipitation pellet from pepsin digestion at 10:1 ratio for times 1 min (S9), 10 min (S10), aggregated list (S11)
- Table S12_S13_S14.xls: BSA peptides identified in precipitation pellet at 100:1 protein to pepsin ratio
- Table S15_S16_ S17: BSA peptides at 1000:1 protein to pepsin ratio.
- Table S18: Amino acid frequency analysis, pellet vs supernatant
- Table S19: Precipitated peptides containing individual residues
- Table S20_S21.xls: List of peptides and proteins identified in the pellet following salt-mediated solvent precipitation of pepsin-digested yeast sample for 2 independent experiments
- Table S22_S23.xls: List of peptides and proteins identified in the pellet following salt-mediated solvent precipitation of pepsin-digested yeast sample for 2 injections to MS
- Table S24: precipitated peptides identified in pellet fraction lacking one or more specific residues

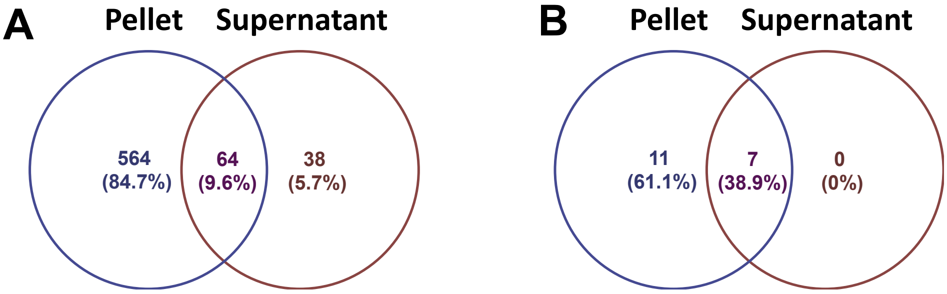

Figure S1. Venn diagram summarizing the proteins identified from bottom-up MS analysis of the pellet and supernatant fractions of (A) yeast and (B) bovine plasma, following peptide precipitation with the optimized protocol.

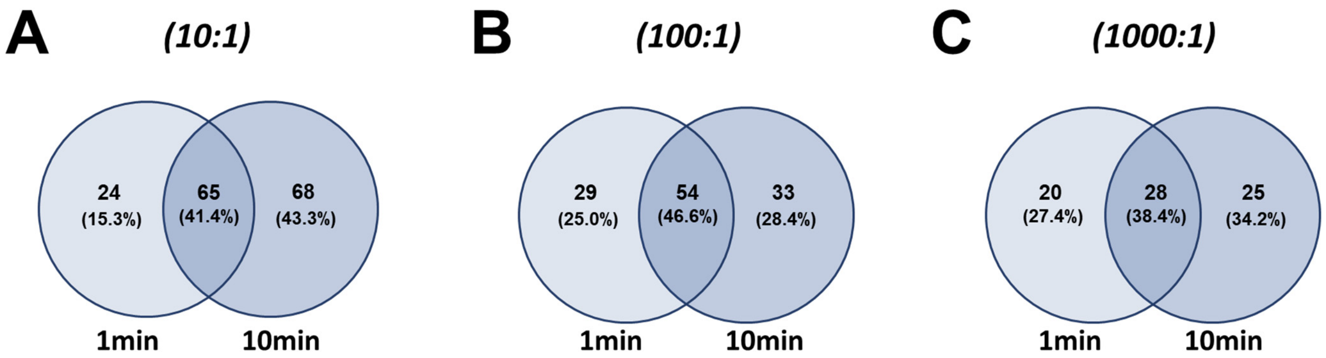

Figure S2. Venn diagrams compare the peptides identified by MS in the pellet fraction following precipitation of BSA, digested with pepsin at various ratios of protein to enzyme: (**a**) 10:1 protein to enzyme ratio; (**b**) 100:1 ratio; (**c**) 1000:1 ratio.

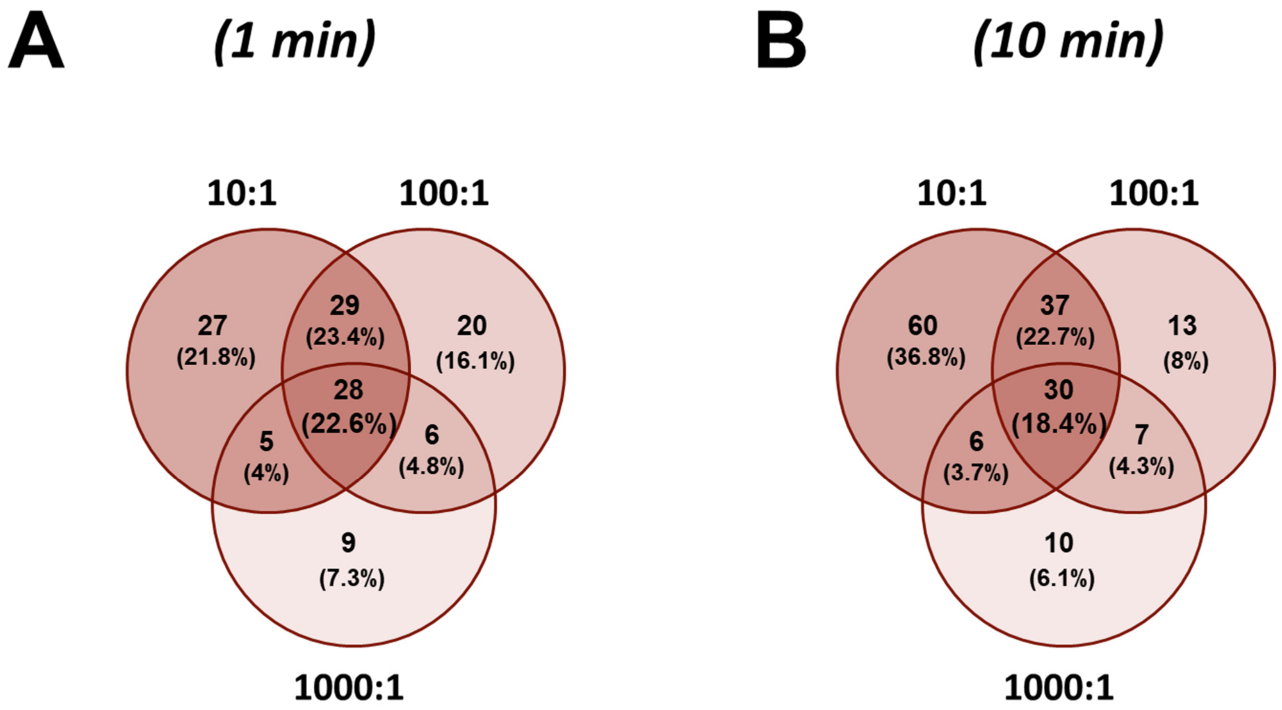

Figure S3. Venn diagrams compare the peptides identified in the pellet fraction following precipitation of BSA, digested with pepsin at various ratios of protein to enzyme for different digestion times: (**a**) 1 min digestion; (**b**) 10 min digestion.

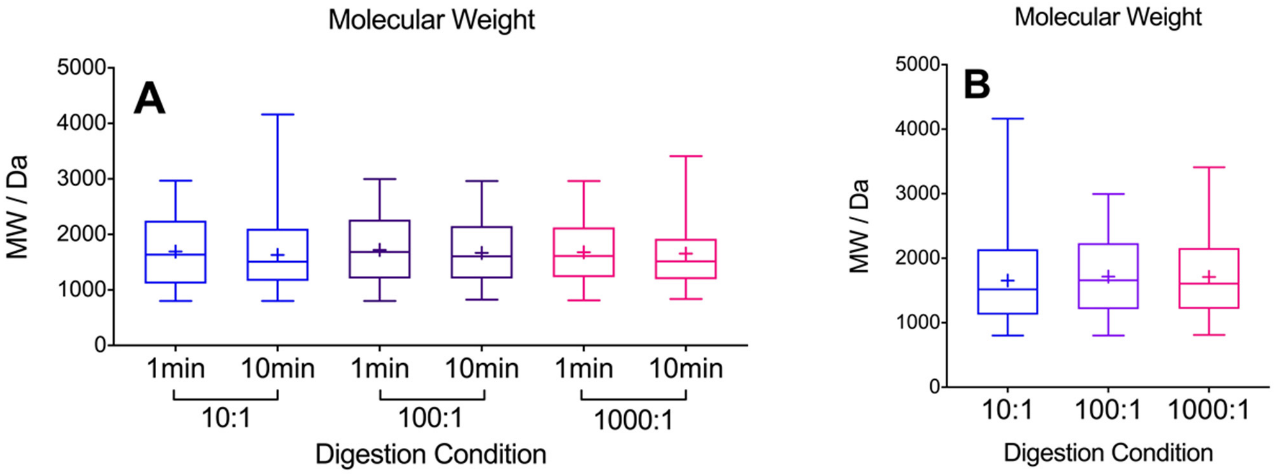

Figure S4. A comparison of the molecular weight distribution of peptides identified by MS following pepsin digestion for specific time (1 vs 10 min) and at different ratios of protein to enzyme (10:1, 100:1, 1000:1). (**a**) Shows that longer digestion times, or more enzyme, relative to protein, yields smaller peptides. (**b**) Summarizes the aggregated set of peptides recovered under the three different ratios of protein to enzyme.

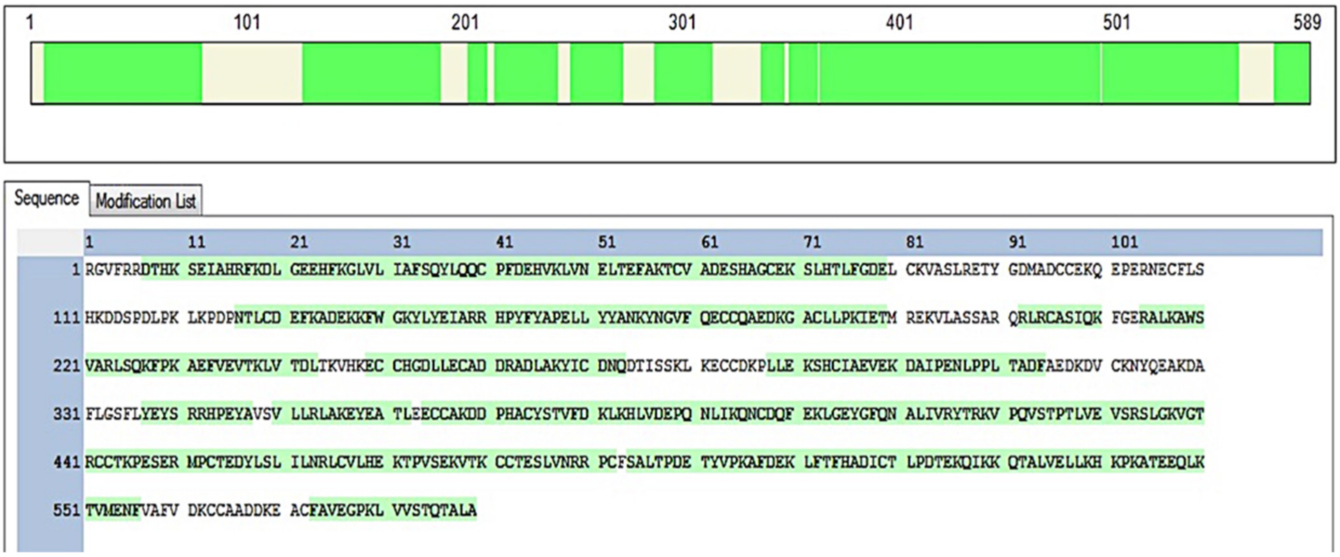

Figure S5. Aggregated sequence coverage obtained from MS analysis of pepsin-digested BSA, with peptides recovered through salt-mediated solvent precipitation.
